# Optimization of Green Synthesis of Silver Nanoparticles from Leaf Extracts of *Pimenta dioica* (Allspice)

**DOI:** 10.1155/2013/362890

**Published:** 2013-12-23

**Authors:** Akshay Rajeev Geetha, Elizabeth George, Akshay Srinivasan, Jameel Shaik

**Affiliations:** ^1^School of Bio Sciences & Technology, VIT University, Vellore 632014, India; ^2^SB Industries, Chennai 600032, India; ^3^Dance with Pal, Bangalore 560075, India

## Abstract

Production of silver nanoparticles from the leaf extracts of *Pimenta dioica* is reported for the first time in this paper. Three different sets of leaves were utilized for the synthesis of nanoparticles—fresh, hot-air oven dried, and sun-dried. These nanoparticles were characterized using UV-Vis spectroscopy and AFM. The results were diverse in that different sizes were seen for different leaf conditions. Nanoparticles synthesized using sun-dried leaves (produced using a particular ratio (1 : 0.5) of the leaf extract sample and silver nitrate (1 mM), resp.) possessed the smallest sizes. We believe that further optimization of the current green-synthesis method would help in the production of monodispersed silver nanoparticles having great potential in treating several diseases.

## 1. Introduction

Among the various inorganic metal nanoparticles, silver nanoparticles have received substantial attention for various reasons—silver is an effective antimicrobial agent that exhibits low toxicity [1, 2]; silver nanoparticles have diverse in vitro and in vivo applications [3, 4]. Although there are many routes [5, 6] available for the synthesis of silver nanoparticles, bioinspired synthesis using plant sources offers several advantages such as cost-effectiveness, eco-friendliness, and the elimination of high pressure, energy, temperature, and toxic chemicals necessary in the traditional synthesis methods [7]. Several plants have been utilized for the production of silver nanoparticles [8–10]. In this work, leaf extracts of *Pimenta dioica* were used for the generation of silver nanoparticles. Numerous studies on *Pimenta dioica* [11, 12] have been conducted and it was found that the leaf extracts generated from *Pimenta dioica* have strong fungicidal [13], bactericidal [14, 15] properties. In addition it also has acaricidal [16, 17], nematicidal [18], anticancer [19], antioxidant [19, 20], and deodorizing properties. All these properties make the leaf extracts generated highly resistant to spoilage and they can be stored for up to three months even in contaminated areas quite safely. However not all leaf extracts from other plant sources stay for so long. This property of *Pimenta dioica* makes it extremely useful for industrial nanoparticle synthesis where long term storage of leaf extract is possible. Besides its use as a spice and flavor, allspice has been used for various gastrointestinal illness, rheumatism, and neuralgia. The leaf extracts also have antiseptic, anesthetic, and several other medical properties [11, 12].

In order to exploit all the above mentioned benefits of bioinspired synthesis along with those provided by nanotechnology, silver nanoparticles were generated from the leaf extracts of *Pimenta dioica* using a previously published method [10]. In addition once we learnt that nanoparticles were synthesised, we determined the optimized conditions for silver nanoparticles synthesis in this plant by studying the influence of different factors on the formation of nanoparticles.

- The first factor we considered for optimization was the leaf sample. We took three variations of leaf extract sample of the plant, that is, fresh, hot-air oven dried, and sun-dried leaves.
- The next factor we considered for optimization is the ratios of mixing of the leaf extract sample and silver nitrate (1 mM). We varied the ratios of mixing of the leaf extract sample and silver nitrate (1 mM) and tested for the ratios 1 : 0.5, 1 : 1, 1 : 2, and 1 : 3 and tried to find out in which case maximum silver nanoparticles were formed.

The UV-Vis spectroscopy and AFM results demonstrated the formation of silver nanoparticles from *Pimenta dioica* extracts. Future studies will involve the applications of the silver nanoparticles generated from the leaf extracts of *Pimenta dioica*.

## 2. Materials and Methods 

### 2.1. Preparation of the Leaf Extracts

Leaf samples were collected from the campus of VIT University and a sample set of the leaves and pulverized leaf samples have been stored for future reference. Three different types of leaf samples were used in the current experiments—fresh leaves, sun-dried leaves, and leaves dried in a hot-air oven (Servewell Instruments Pvt. Ltd., Bangalore, India) at 60°C for two hours. Several researchers have reported virtually no beneficial results from sun-dried leaves. But since there are individual differences in the phytochemicals' contents in different plants and also as to not exclude any unexpected good and beneficial results, we included sun-dried leaves in our previous study [10] and plan to include them in all our future studies too.

The procedures adapted for all the above-mentioned samples were the same. In the case of sample one, 25 grams of the leaves were thoroughly washed three times in distilled water for 15 min, air dried, cut into fine pieces, and were boiled in a Erlenmeyer flask with 150 mL of sterile distilled water for 5 min and were finally filtered to get the leaf extract. For samples two and three, 25 grams of the leaves were dried, pulverized, and the procedure used for sample one was followed.

### 2.2. Synthesis of Silver Nanoparticles

Leaf extract of each sample was added into the aqueous solution of 1 mM silver nitrate (AgNO_3_, MW 169.87, SISCO Research Laboratories Pvt. Ltd., Mumbai, India) in four different ratios 1 : 0.5, 1 : 1, 1 : 2, and 1 : 3 (by volume). Three sample solutions in four ratios (fresh leaves (FL), hot-air oven dried (OD) leaves, and sun-dried (SD)) along with the silver nitrate solution were centrifuged at 12000 rpm for 15 minutes. Thus, a total of 12 different samples were obtained:

 FL 1 : 0.5—fresh leaf extract in ratio 1 : 0.5 with AgNO_3_ solution;
 FL 1 : 1—fresh leaf extract in ratio 1 : 1 with AgNO_3_ solution;
 FL 1 : 2—fresh leaf extract in ratio 1 : 2 with AgNO_3_ solution;
 FL 1 : 3—fresh leaf extract in ratio 1 : 3 with AgNO_3_ solution;
 OD 1 : 0.5—oven dried leaf extract in ratio 1 : 0.5 with AgNO_3_ solution;
  OD 1 : 1—oven dried leaf extract in ratio 1 : 1 with AgNO_3_ solution;
  OD 1 : 2—oven dried leaf extract in ratio 1 : 2 with AgNO_3_ solution;
  OD 1 : 3—oven dried leaf extract in ratio 1 : 2 with AgNO_3_ solution;
  SD 1 : 0.5—sun-dried leaf extract in ratio 1 : 0.5 with AgNO_3_ solution;
  SD 1 : 1—sun-dried leaf extract in ratio 1 : 1 with AgNO_3_ solution;
  SD 1 : 2—sun-dried leaf extract in ratio 1 : 2 with AgNO_3_ solution;
  SD 1 : 3—sun-dried leaf extract in ratio 1 : 3 with AgNO_3_ solution.

All the three samples of *Pimenta dioica* were prepared in triplicate for all the conditions.

### 2.3. Spectrophotometric Analysis of the Samples

An Ultrospec 1100 Pro UV/Visible Spectrophotometer (Amersham Biosciences) was used for the spectrophotometric analysis. The reduction of pure silver ions was monitored by measuring the UV-Vis spectrum of the colloidal solution obtained after 10 min of adding 300 *μ*L of sample solution to 3 mL of deionized water. A graph of wavelength on *x*-axis and absorbance on *y*-axis was plotted.

### 2.4. AFM Analysis of Silver Nanoparticles

The silver nanoparticles extracted by the above protocol were visualized with an atomic force microscope (AFM). A thin film of the sample was prepared on a glass slide by dropping 100 *μ*L of the sample on the slide and was allowed to dry for 5 min. The slides were then scanned with the AFM (Nanosurf AG, Switzerland, Product: BT02089, v1.3R0). Nanosurf Easyscan-2 software was used for the AFM analysis.

## 3. Results

The current study was undertaken to exploit the hitherto un-utilized plant sources in the development of silver nanoparticles. The plant *Pimenta dioica* belonging to the family Myrtaceae was selected and used in this first ever report on the synthesis of silver nanoparticles from its leaf extracts and to determine the optimized conditions for silver nanoparticle synthesis in this plant.

**Figure 1:**
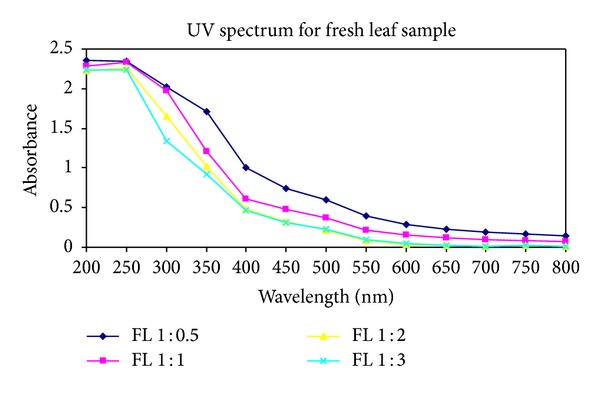
UV-Vis absorption spectra recorded as a function of time of reaction of four different solutions of silver ions by *Pimenta dioica* fresh leaves extract in ratios of 1 : 0.5, 1 : 1, 1 : 2, and 1 : 3 after 10 minutes reaction kinetics.

**Figure 2:**
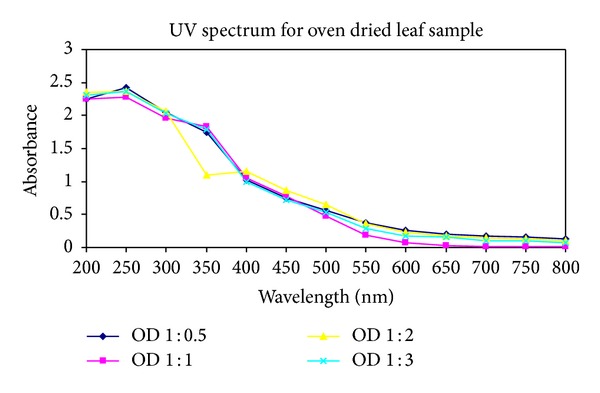
UV-Vis absorption spectra recorded as a function of time of reaction of four different solutions of silver ions by *Pimenta dioica* oven-dried leaves extract in ratios of 1 : 0.5, 1 : 1, 1 : 2, and 1 : 3 after 10 minutes reaction kinetics.

**Figure 3:**
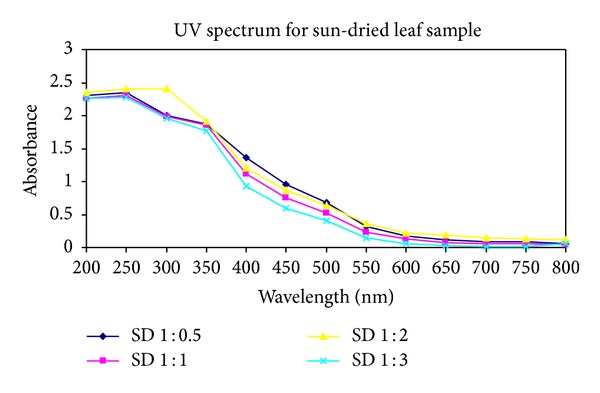
UV-Vis absorption spectra recorded as a function of time of reaction of four different solutions of silver ions by *Pimenta dioica* sun-dried leaves extract in ratios of 1 : 0.5, 1 : 1, 1 : 2, and 1 : 3 after 10 minutes reaction kinetics.

After the extraction of silver nanoparticles, UV-Vis spectrophotometric measurements were performed for all twelve samples obtained from the first three. The reduction of silver ions in the aqueous solution of silver complex during the reaction with the ingredients of the leaf extracts revealed that silver nanoparticles in the solution could be correlated with the respective UV-Vis spectra. Using spectrophotometric analysis, the colloidal solution of each sample studied exhibited a strong absorption between 250 and 450 nm (see Figures 1, 2, and 3).

**Figure 4:**
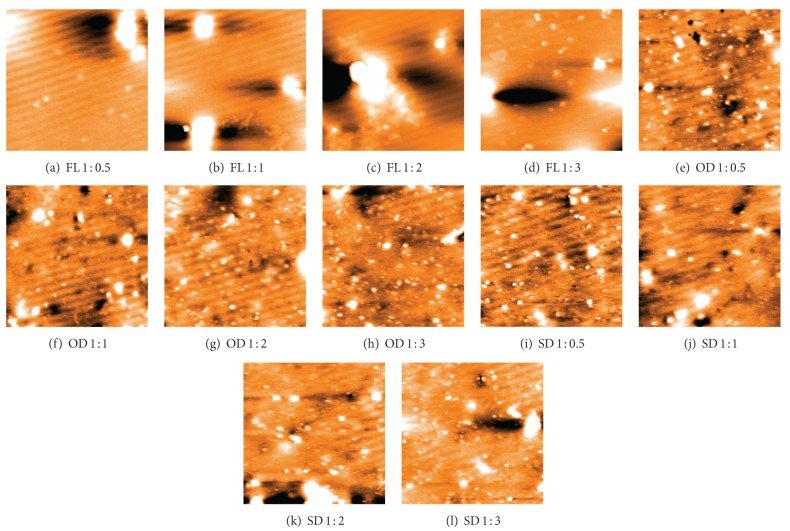
AFM micrographs of silver nanoparticles synthesized from the leaf extracts of *Pimenta dioica*: (a) fresh leaves in 1 : 0.5 ratio with silver nitrate, (a) fresh leaves in 1 : 1 ratio with silver nitrate, (a) fresh leaves in 1 : 2 ratio with silver nitrate, and (a) fresh leaves in 1 : 3 ratio with silver nitrate; (b) sun-dried leaves in 1 : 0.5 ratio with silver nitrate, (b) sun-dried leaves in 1 : 1 ratio with silver nitrate, (b) sun-dried leaves in 1 : 2 ratio with silver nitrate, and (b) sun-dried leaves in 1 : 3 ratio with silver nitrate (c) hot-air oven dried leaves in 1 : 0.5 ratio with silver nitrate (c) hot-air oven dried leaves in 1 : 1 ratio with silver nitrate; (c) hot-air oven dried leaves in 1 : 2 ratio with silver nitrate and (c) hot-air oven dried leaves in 1 : 3 ratio with silver nitrate.

**Figure 5:**
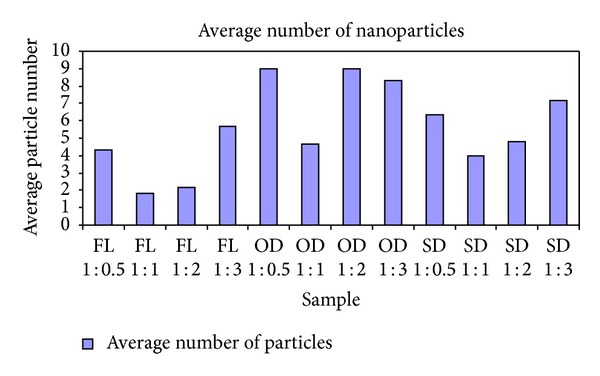
Average number of silver nanoparticles synthesized from the leaf extracts of *Pimenta dioica* under different conditions.

**Figure 6:**
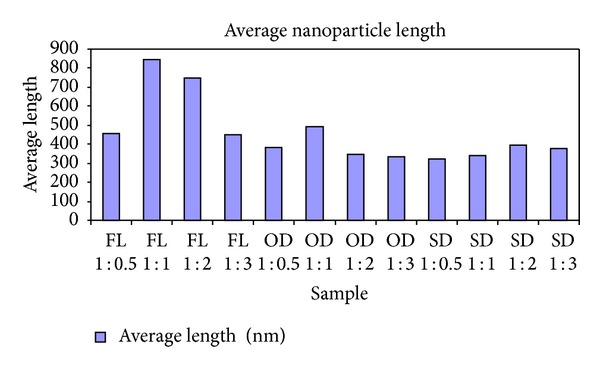
Average length of silver nanoparticles synthesized from the leaf extracts of *Pimenta dioica* under different conditions.

**Figure 7:**
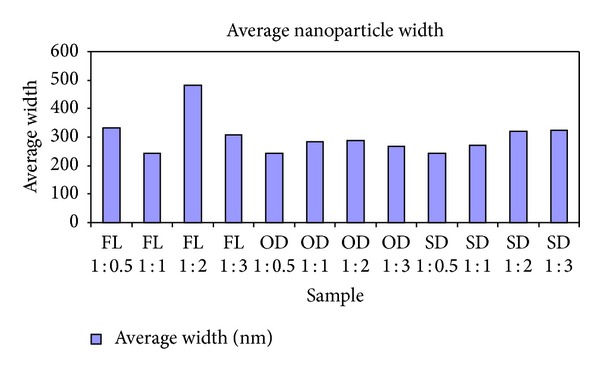
Average width of silver nanoparticles synthesized from the leaf extracts of *Pimenta dioica* under different conditions.

The silver nanoparticles extracted from twelve samples (three different leaf extracts in four ratios) were also analyzed using AFM.  Figure 4 contains the micrographs obtained from the AFM measurements of the silver nanoparticles obtained under different conditions using *Pimenta dioica* leaf extracts. All the figures—Figures 4(a)–4(l)—illustrate an AFM micrograph of silver nanoparticles synthesized from fresh leaf extracts scanned in an area of 15 *μ*m × 14.9 *μ*m. Figures 5, 6, and 7 contain the average lengths (measured along the longer axis of nanoparticles), widths (measured along the shorter axis of nanoparticles), and areas calculated for the silver nanoparticles obtained under different conditions of the leaves.

**Figure 8:**
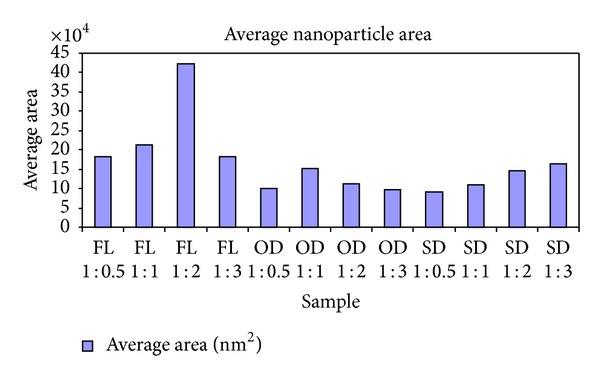
Average area of silver nanoparticles synthesized from the leaf extracts of *Pimenta dioica* under different conditions.

Twelve different AFM micrographs of equal areas were obtained for the three different leaf conditions in four ratios and average number of nanoparticles, average lengths, widths, and areas of the nanoparticles were calculated in these areas (Figures 5–8).

## 4. Discussion

By analysing these graphs, we observed that the silver nanoparticles prepared from the extracts of sun-dried leaves in the ratio 1 : 0.5 (SD 1 : 0.5) possessed the smallest sizes, while the largest sizes were possessed by the silver nanoparticles from fresh leaf samples in the ratio 1 : 1 (FL 1 : 1) and the sizes of the silver nanoparticles obtained from the hot-air oven dried leaf samples in ratio 1 : 1 (OD 1 : 1) were in between the sizes of the nanoparticles obtained under the other two conditions.

In addition, it was found that the greatest numbers of silver nanoparticles were formed from the hot-air oven dried leaf samples in ratios 1 : 1 (OD 1 : 1) and 1 : 2 (OD 1 : 2). Hot-air oven dried leaf samples in ratio 1 : 3 (OD 1 : 3) also gave high values of silver nanoparticles. The least number of nanoparticles was obtained from fresh leaf samples in the ratio 1 : 1 (FL 1 : 1).

It was also found that the leaf sample dried in hot-air oven in ratio of 1 : 2 (OD 1 : 2) showed the best results when viewed under the AFM as the nanoparticles produced were in high number and in small sizes.

## 5. Conclusions

It is clear from the above results that the *Pimenta dioica* leaf extracts processed under different conditions are highly promising in the development of silver nanoparticles of different sizes of nanoparticles that could be tailored for specific applications. However, the processing conditions need to be optimized further to extract spherical and monodispersed nanoparticles.
